# Emergence of physiological rhythmicity in term and preterm neonates in a neonatal intensive care unit

**DOI:** 10.1186/1740-3391-4-11

**Published:** 2006-09-11

**Authors:** Esmot ara Begum, Motoki Bonno, Makoto Obata, Hatsumi Yamamoto, Masatoshi Kawai, Yoshihiro Komada

**Affiliations:** 1 Clinical Research Institute and Department of Pediatrics, National Hospital Organization, Miechuo Medical Center, 2158-5 Hisai Myojin Cho, Tsu City, Mie 514, Japan; 2 Department of Developmental Clinical Psychology, Institute for Education, Mukogawa Women's University, 6-46 Ikebiraki Cho, Nishinomiya City, Hyogo 633, Japan; 3 Department of Pediatrics and Developmental Science, Mie University Graduate School of Medicine, 174-2 Edobashi, Tsu City, Mie 514, Japan

## Abstract

**Background:**

Biological rhythmicity, particularly circadian rhythmicity, is considered to be a key mechanism in the maintenance of physiological function. Very little is known, however, about biological rhythmicity pattern in preterm and term neonates in neonatal intensive care units (NICU). In this study, we investigated whether term and preterm neonates admitted to NICU exhibit biological rhythmicity during the neonatal period.

**Methods:**

Twenty-four-hour continuous recording of four physiological variables (heart rate: HR recorded by electrocardiogram; pulse rate: PR recorded by pulse oxymetry; respiratory rate: RR; and oxygen saturation of pulse oxymetry: SpO_2_) was conducted on 187 neonates in NICU during 0–21 days of postnatal age (PNA). Rhythmicity was analyzed by spectral analysis (SPSS procedure Spectra). The Fisher test was performed to test the statistical significance of the cycles. The cycle with the largest peak of the periodogram intensities was determined as dominant cycle and confirmed by Fourier analysis. The amplitudes and amplitude indexes for each dominant cycle were calculated.

**Results:**

Circadian cycles were observed among 23.8% neonates in HR, 20% in PR, 27.8% in RR and 16% in SpO_2 _in 0–3 days of PNA. Percentages of circadian cycles were the highest (40%) at <28 wks of gestational age (GA), decreasing with GA, and the lowest (14.3%) at >= 37 wks GA within 3 days of PNA in PR and were decreased in the later PNA. An increase of the amplitude with GA was observed in PR, and significant group differences were present in all periods. Amplitudes and amplitude indexes were positively correlated with postconceptional age (PCA) in PR (p < 0.001). Among clinical parameters, oxygen administration showed significant association (p < 0.05) with circadian rhythms of PR in the first 3 days of life.

**Conclusion:**

Whereas circadian rhythmicity in neonates may result from maternal influence, the increase of amplitude indexes in PR with PCA may be related to physiological maturity. Further studies are needed to elucidate the effect of oxygenation on physiological rhythmicity in neonates.

## Background

Preterm neonates hospitalized in a neonatal intensive care unit (NICU) face many challenges to adapt to the new environment. Heat loss [1], weight loss [2], respiratory distress and cardiac instability [3] are very common features for them. An artificial environment in NICU is mandatory to support these neonates; however, external influences such as constant light, noise, and medical intervention may be stressful. Further, neonates are deprived of maternal influences, which is essential for their development. It has been thought that this environmental condition may influence the development of biological rhythm in preterm neonates [4-6].

Circadian rhythms are generated endogenously by a biological clock, which is located in the anterior hypothalamic suprachiasmatic nuclei (SCN) [7,8], and are modulated by exogenous factors [9,10]. Many physiological processes are now known to be cyclically organized [11]. They show different cycles: circadian cycles last approximately 24 hours, ultradian cycles shorter than 24 hours, and infradian cycles longer than 24 hours [12]. These rhythms interact mutually as well as with the outside fluctuating environment under the control of feedback systems providing an orderly function that enables life [11].

Circadian rhythms have been described in the human fetus [13-16] and have been attributed either to the maternal environment or to the maturation of the fetal nervous system [13,17,18]. The SCN has been detected as early as 18–20 weeks of gestational age [19], and primate studies indicated that the SCN is responsive to light at 24 weeks of gestational age [20]. In term neonates, circadian rhythms have been reported to be present immediately after birth but to eventually disappear [4,21], not being detected again until 3 to 4 weeks of postnatal life [22]. Some studies showed that circadian rhythms are predominant in preterm neonates [4,21,23], while others showed ultradian rhythms to be dominant in preterm neonates [22,24-27]. To elucidate the developmental process of physiological rhythmicities, we studied four physiological variables in preterm and term neonates.

## Methods

### Subjects and data collection

From January 2004 to March 2006, 520 neonates were admitted to the NICU at Miechuo Medical Center. All of them were monitored with electrocardiogram (ECG) for heart rate (HR), respiration rate (RR), and with pulse oxymetry on the wrist or the feet for saturation of pulse oxymetry oxygen (SpO_2_) and pulse rate (PR) throughout their stay in the NICU. Monitored physiological information was transformed as measurement variables at 10-second intervals by the Wave Achieving System (WAS-J; Philips Electronics Japan, Tokyo, Japan) through the local area network in the NICU. The data were recorded for 24 hours for the following postnatal periods: Period 1: days 0–3; Period 2: days 4–6; Period 3: days 7–13; and Period 4: days 14–21. Subjects with continuously disrupted data for more than 1 minute were excluded from the study. A total of 187 neonates (114 boys and 73 girls) were recorded from period 1 to period 4.

The NICU was maintained under a light-dark cycle. The light was dimmed (less than 30 lux) during the night from 21:00 pm to 07:00 am, while it was maintained at a higher level (300–580 lux) during the daytime. NICU staff also varied according to time of day: the number of attendants at night was one third that of attendants during daytime hours. Parent's visitations were allowed three times a day (11:00 to 12:00 in the morning, 14:00 to 15:00 in the afternoon, and 17:00 to 21:00 in the evening). Bathing and measurement of body weight were conducted daily in the morning. Medical examinations, such as blood sampling, radiography, or ultrasonography, were mostly provided in the morning if necessary.

Written informed consent was obtained from the parents, and the study was approved by the ethical committee of the institute. Demographics and health status information's were obtained from the medical records.

### Analysis of rhythms

**Figure 1:**
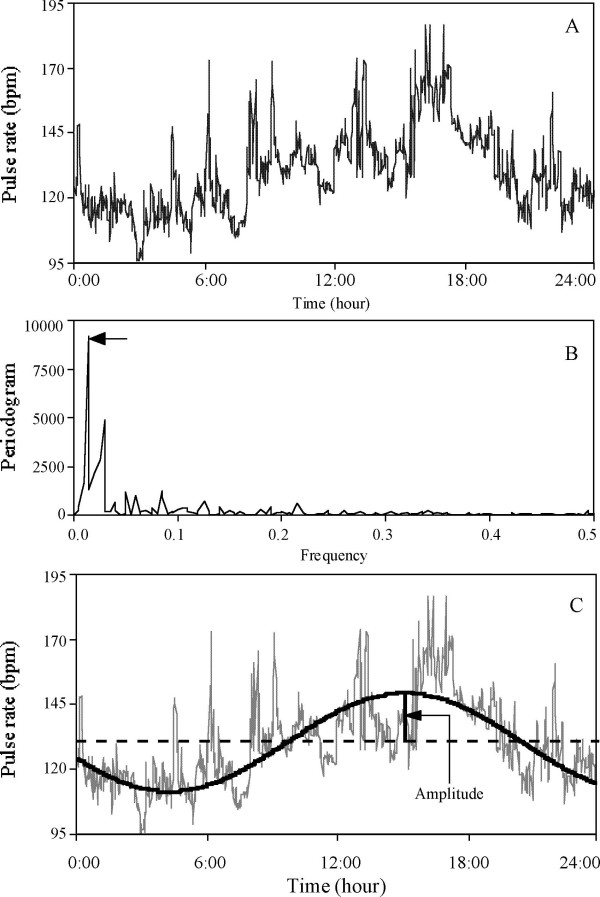
**Brief description of steps to determine the dominant cycle using spectral analysis**. ***A***: Plot of original data for pulse rate (PR). PR was measured once every 10 seconds and averaged into 1 minute time block for 1440 minutes; N = 1440 observation. ***B***: Periodogram intensities for PR (plotted on linear scale). The largest peak of the periodogram was selected (arrow) as representative cyclic component that represent the largest amount of variance. ***C***: The corresponding cycle of the largest peak in the periodogram intensities was reconstructed from the FFT coefficient to fit the sinusoidal function: *χ*_t _= *μ *+ Acos(*ω *t) + Bsin(*ω *t). The bold line is the detected cycle (period: 1440 minutes = 24 hours) superimposed on the original data.

Physiological rhythmicity was analyzed for HR, PR, RR and SpO_2 _with spectral analysis (periodogram) with SPSS 11.5 software (SPSS Inc. Chicago, IL), as previously reported [28]. Briefly, 24 hours sessions were run in 10-second intervals and were aggregated into 1-minute time blocks. Periodogram analysis was performed with a time series of 1440 minutes (N = 1440 observations). The Fisher test was used to test the statistical significance of the cyclic components (N = 1440, α = 0.05) [28,29]. Among the significant cycles, the cycle with the largest peak in the periodogram was considered to be the dominant cycle for each time series data and was used for further analysis [28]. All dominant cycles were confirmed by Fourier analysis, and further circadian cycles were confirmed by cosinor analysis with a significance of p < 0.05 by least square analysis (Figure 1). The amplitude, the distance between mesor and the highest value of the cosine curve, was calculated for each dominant cycle. In addition, an amplitude index was calculated as follows:

Amplitude index = amplitude ÷ mean of variables × 100.

### Statistical analysis

Data were analyzed with SPSS and Statview. ANOVA was used to evaluate the differences between gestational age groups. The Pearson correlation coefficient was used to analyze the relationships between postconceptional age (PCA) and rhythmicity parameters. Univariate analysis using Mann-Whitney U-test for continuous variables or Fisher's exact test for categorical variables was used to compare clinical variables according to the development of physiological rhythmicity. A multiple logistic regression analysis was performed using a step-wise approach to determine the independent relationship of significant variables found in the univariate analysis.

## Results

### Sample characteristics

The demographics of neonates are shown in Table 1. The median gestational age (GA) was 34 weeks (range: 23–42 weeks), and the median birth weight was 1968 g (range: 454–4132 g). Among these neonates, 9.1% were born at < 28 weeks of gestation age and 14.4% had birth weight of less than 1000 g. The median age at hospitalization was 0 day (range: 0–9 day) and the median duration of hospitalization was 32 days (range: 5–182 days). One hundred eleven neonates (59.4%) were intubated and 72 neonates (38.5%) received oxygen.

**Table 1:** Demographic characteristics of 187 preterm and term neonates.

***Variables/Categories***	***n (%)***
Gender (boys/Girls)	114 (61)/73 (39)
Gestational age (wks), median (range)	34 (23–42)
< 28	17 (9.1)
28–32	49 (26.2)
33–36	58 (31)
≥37	63 (33.7)
Birth Weight (g), median (range)	1968 (454–4132)
< 1000	27 (14.4)
1000–1499	31 (16.6)
1500–1999	38 (20.3)
≥2000	91 (48.7)
Apgar score 1 min/5 min, median (range)	8 (0–10)/9 (2–10)
Age at hospitalization (day), median (range)	0 (0–9)
Hospitalization (day), median (range)	32 (5–182)
Caesarian Section	96 (51.3)
Multiple gestation	4 (2.3)
Intubation	111(59.4)
Oxygenation	72 (38.5)
Birth asphyxia	27 (14.4)
Intrauterine growth retardation	23 (12.3)
Respiratory distress syndrome	31 (16.6)
Transient tachypnea of the newborn	38 (20.3)

Data are expressed as mean ± SD or n (%).

### Rhythmicity analysis

Results of the analyses of rhythmicity are summarized in Table 2. To ensure the accuracy of rhythmicity analysis, parameters missing more than 7% of total data were excluded from the analysis in each study. Among 461 time series recorded for each parameters, eligible samples were obtained in 304 for HR, 379 for PR, 372 for RR, and 383 for SpO_2 _within the 4 periods. Among eligible samples, rhythmicity was observed in more than 90% of neonates in each period for HR, PR, RR and SpO_2 _(Table 2). The percentage was not much lower (HR: 89%, PR: 90%, RR: 79%, SpO_2_: 76%) after Bonferroni correction for multiple testing (p < 0.0001).

**Table 2:** Descriptive profiles for significant cycles of HR, PR, RR and SpO_2_.

Period		Period 1	Period 2	Period 3	Period 4
Sampling		(0–3)	(4–6)	(7–13)	(14–21)
n		116	114	125	106
Eligible sample*	HR	82 (70.7)	64 (56.1)	91 (72.8)	67 (63.2)
	PR	101 (87.1)	88 (77.2)	106 (84.8)	84 (79.2)
	RR	99 (85.3)	85 (74.6)	104 (83.2)	84 (79.2)
	SpO2	103 (88.8)	89 (78.1)	106 (84.8)	85 (80.2)
Significant cycle**	HR	80 (98)	64 (100)	89 (98)	67 (100)
	PR	100 (99)	87 (99)	104 (98.1)	83 (99)
	RR	90 (91)	84 (99)	97 (93.3)	79 (94)
	SpO2	94 (91.3)	86 (97)	103 (97)	78 (92)
Circadian cycle***	HR	19 (23.8)	11 (17.2)	20 (22.5)	13(19.4)
	PR	20 (20)	16 (18.4)	20 (19.2)	16 (19.3)
	RR	25 (27.8)	28 (33.3)	21 (21.6)	11 (13.9)
	SpO2	15 (16)	10 (11.6)	17 (16.5)	15 (19.2)

Data are shown in n (%). Parentheses are percentages of * eligible samples in all samples, ** significant cycles in all eligible samples, and *** circadian cycles in significant cycles.

Without correction for multiple testing, circadian cycle (1440 minutes) was observed among 23.8% neonates in HR, 20% in PR, 27.8% in RR and 16% in SpO_2 _in Period 1. Because many samples were excluded from HR analysis, and the percentage of eligible samples was consistently lower than for PR, further analysis of cardiac rhythmicity used PR instead of HR.

### Rhythmicity and gestational age

Rhythmicity was analyzed in four gestational age groups: < 28 wks, 28–32 wks, 33–36 wks, ≥ 37 wks. The distribution of circadian cycles in each gestational age groups and periods is summarized in Table 3. In PR, the percentage of circadian cycles was highest (40%) at <28 wks of GA, decreasing with GA, and lowest (14.3%) at ≥ 37 wks of GA in Period 1. A similar tendency was observed in each period in PR; however, there was no consistent tendency in percentages of circadian cycle in RR and SpO_2_.

**Table 3:** Distribution of circadian cycles according to gestational age groups in each period.

	Gestational age		Period 1		Period 2		Period 3		Period 4
	Groups	n	(0–3 d)	n	(4–6 d)	n	(7–13 d)	n	(14–21 d)
PR	<28 wks	10	4 (40)	12	3 (25)	12	5 (41.7)	13	4 (30.8)
	28–32 wks	26	6 (23.1)	22	6 (27.3)	42	11 (26.2)	39	9 (23.1)
	33–36 wks	29	5 (17.2)	26	5 (19.2)	31	2 (6.5)	23	3 (13.0)
	≥37 wks	35	5 (14.3)	27	2 (7.4)	19	2 (10.5)	8	0 (0)
RR	< 28 wks	7	1 (14.3)	11	1(9.1)	13	5 (38.5)	13	0 (0)
	28–32 wks	24	8 (33.3)	20	9 (45)	38	9 (23.7)	36	8 (22.2)
	33–36 wks	25	8 (32)	27	9 (33.3)	28	3 (10.7)	22	2 (9.1)
	≥37 wks	34	8 (23.5)	26	9 (34.6)	18	4 (22.2)	8	1(12.5)
SpO2	< 28 wks	10	0 (0)	12	3 (25)	12	3 (25)	13	2 (15.4)
	28–32 wks	25	5 (20)	20	3 (15)	40	7 (17.5)	37	9 (24.3)
	33–36 wks	26	5 (19.2)	25	5 (20)	32	4 (12.5)	20	3 (15)
	≥37 wks	33	5 (15.2)	29	4 (13.8)	19	3 (15.8)	8	1 (12.5)

Data are shown in n (%).

**Figure 2:**
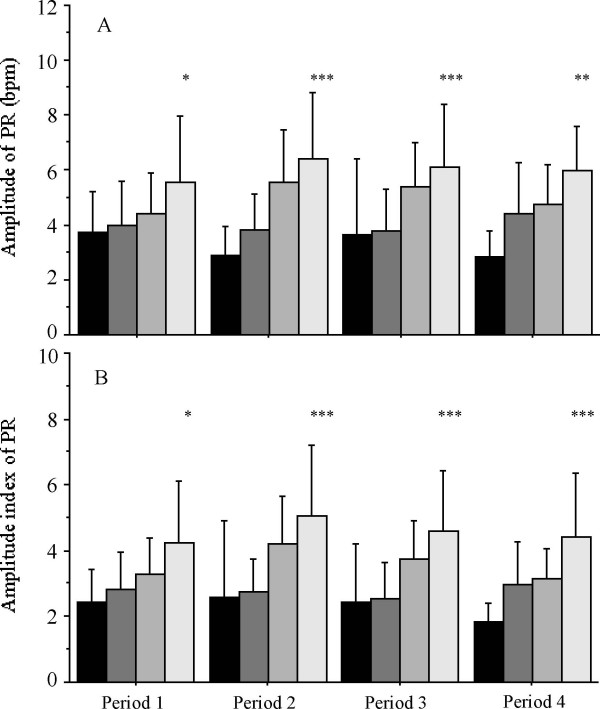
**Amplitudes (*A*) and amplitude indexes (*B*) of all detected cycle of PR over the 4 periods for 4 gestational age groups infants**. Data are shown in Mean ± SD. The dark bar is for < 28 wks, the gray bar is for 28–32 wks, the light gray bar is for 33–36 wks, and white bar is for ≥ 37 wks. * p < 0.01, ** p < 0.001, *** p < 0.0001, according to ANOVA. The sample size for each gestational age group is shown in Table 2.

Amplitudes and amplitude indexes of all detected cycles in PR in each period are shown in Figure 2. An increase of circadian amplitude with gestational age was observed in PR. and significant differences were present among gestational age groups in all periods (Figure 2A). These changes were not observed in RR and SpO_2 _(data not shown). Amplitude indexes showed similar tendency to amplitudes in PR (Figure 2B). There were no significant associations between cycles and amplitudes in any parameter in each period (data not shown).

### Relationship between rhythmicity and postconceptional age

**Figure 3:**
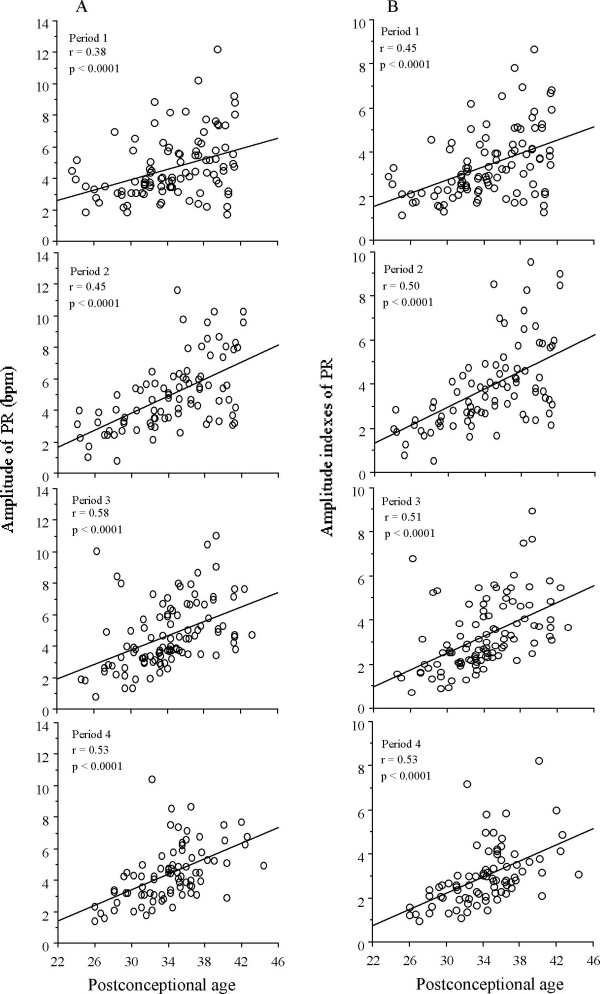
**Linear regression (and coefficients of correlation) for amplitudes and amplitude indexes of PR as functions of postconceptional age**. A significant increase in amplitudes and amplitude indexes with postconceptional age is present in all period in PR.

In examining the relationship with postconceptional age (PCA), correlation of coefficient was performed using amplitudes and amplitude indexes in each period for all parameters. Amplitudes and amplitude indexes of PR were positively correlated with PCA in all four periods (Figure 3).

### Clinical conditions associated with rhythmicity

To determine whether clinical conditions may affect the emergence and development of rhythmicity, clinical factors were determined according to cycle length with circadian cycles (1440 minutes) or ultradian cycles (≤ 720 minutes). On univariate analyses in Period 1, circadian cycle (1440 minutes) was significantly associated (p < 0.05) only with oxygen administration at data sampling in PR (Table 4), while there were no significant associations in RR or SpO_2 _(data not shown). In Periods 3 and 4 in PR, gestational age was found to be significantly associated with circadian cycle (p < 0.01) as well as with oxygen administration (p < 0.05). Neither gestational age nor oxygen administration qualified as an independent factor for existence of circadian cycle in multivariate logistic regression models. Clinical parameters were not associated with the existence of significant cycles in amplitude or amplitude index.

**Table 4:** Univariate analysis for association of clinical parameters with existence of circadian rhythmicities in PR in Period 1.

Clinical variables	Cycle 1440 (n = 20)	≤ 720 (n = 80)	p
Gestational age (wks)	32.7 ± 4.9	34.2 ± 4.6	NS
Birth weight (g)	1930 ± 983	2077 ± 900	NS
Apgar Score < 6 (5 min)	1 (5)	10 (12.7)	NS
Asphyxia	4 (20)	17 (21.3)	NS
RDS	4 (20)	14 (17.3)	NS
IUGR	3 (15)	6 (7.5)	NS
Mean of variables			
Mean PR (/min)	140.2 ± 8.6	135.5 ± 12.8	NS
Mean RR (/min)	45.7 ± 8.5	43.0 ± 8.5	NS
Mean SpO2 (%)	97.9 ± 1.1	97.9 ± 1.3	NS
Treatment of data sampling			
Oxygenation	18 (90)	46 (57.5)	0.02
Intubation	10 (50)	25 (31.3)	NS
Aminophylline	1 (5)	4 (5)	NS
Phenobarbital	0 (0)	1 (1.3)	NS
Midazolam	3 (15)	6 (7.5)	NS

Data are expressed as mean ± SD or n (%). Mann-Whitney U test was performed for continuous variables and Fisher's exact test was performed for categorical variables.

## Discussion

Rhythmicity has been previously studied in preterm and term infants for various physiological variables, such as body temperature [24,30], blood pressure [21], heart rate [18], sleep-wake pattern [24], rest-activity pattern [26], melatonin secretion [31], and electroencephalogram [32]. In this study, we have investigated rhythmicity in PR, RR, and SpO_2_. All of these are important parameters in the regulation of human physiology, and yet little is known about rhythmicity of these variables in neonates. We have shown that most of the analyzed neonates had individual rhythmicity for these parameters with variable cycles after birth, even in extremely immature infants.

Emergence of circadian rhythmicity has been reported to be associated with brain maturation of preterm infants [33,34]. In term neonates, circadian cycles are detected immediately after birth and subsequently disappear and are not detectable until 3 to 4 weeks of postnatal life [22]. It has been suggested that circadian cycles in the early neonatal period are due to maternal influence in utero and that endogenous rhythmicity appears only later [13,17,18]. However, conclusive studies are limited by subject number because of the difficulty in collecting continuous data in NICU. Our sample size of 187 neonates is larger than that of previous studies. As a result, circadian cycles were confirmed in early neonatal period for all parameters either in preterm or term neonates. In PR, comparatively higher percentages of circadian cycles were observed during early neonatal period in preterm neonates and persisted through the later neonatal period, especially in extremely immature infants, while percentages of circadian cycles decreased through the later period in term neonates. These results partially support the previous studies [4,21,23]. The fact that environmental conditions were rhythmic in our study (i.e., presence of a light-dark cycle, of a cycle of NICU staffing, of a cycle of bathing, etc.) prevents us from making inferences about the endogenous or exogenous nature of biological rhythmicity in our subjects.

Although exact factors for the development of rhythmicity are still unclear, it has been suggested that physiological complications may play a role [35]. Among clinical parameters, disease conditions such as respiratory problems or asphyxia, and therapeutic drugs such as phenobarbital or aminophylline, were not associated with emergence of circadian cycles. Only oxygen administration revealed significant association with emergence of circadian cycles in PR within 3 days of birth. Disruption of circadian rhythmicity by reduction of oxygen supply, and restoration by re-oxygenation, has been demonstrated in rats [36,37]. Reduced oxygen activates hypoxia-inducible factor 1(HIF-1) [38], which is involved in oxygen homeostasis. Chilov and colleagues also indicated that oxygen supply modulates the circadian clock at the molecular levels via HIF-1 in the mouse brain [39]. Our observations support these experimental results and suggested that oxygen supply may also influence rhythmicity in humans. Further analyses are required to explore the influencing mechanisms on emergence of rhythmicities in neonates.

## Conclusion

Preterm neonates are at great risk of life-threatening events such as infection, respiratory distress or circulatory failure. As shown in this study, co-existence of circadian cycles with low amplitude in preterm neonates may complementarily support immature homeostasis and function against unstable physiological condition. Our results should aid further research on physiological rhythmicity in neonates.

## Competing interests

The author(s) declare that they have no competing interests.

## Authors' contributions

EB and MB participated in all data collection, in the analysis and discussion of the results, and in the writing of the manuscript. MO participated in clinical data collection and advised on clinical implications of physiological rhythmicity. HY established the NICU local area network system (NICU LAN system) for physiological data recording and advised on clinical implications of physiological rhythmicity. MK provided advice on neonatal physiology and physiological rhythmicity. YK organized the study group, obtained grant support, and supervised the writing of the manuscript. All authors read and approved the final manuscript.

## References

[B1] Hammarlund K, Stromberg B, Sedin G (1986). Heat loss from the skin of preterm and fullterm newborn infants during the first weeks after birth. Biol Neonate.

[B2] Bauer K, Versmold H (1989). Postnatal weight loss in preterm neonates less than 1,500 g is due to isotonic dehydration of the extracellular volume. Acta Paediatr Scand Suppl.

[B3] Di Fiore JM, Arko MK, Miller MJ, Krauss A, Betkerur A, Zadell A, Kenney SR, Martin RJ (2001). Cardiorespiratory events in preterm infants referred for apnea monitoring studies. Pediatrics.

[B4] Mirmiran M, Kok JH (1991). Circadian rhythms in early human development. Early Hum Dev.

[B5] Rivkees SA (2003). Developing circadian rhythmicity in infants. Pediatrics.

[B6] Mirmiran M, Kok JH, Boer K, Wolf H (1992). Perinatal development of human circadian rhythms: role of the foetal biological clock. Neurosci Biobehav Rev.

[B7] Panda S, Hogenesch JB, Kay SA (2002). Circadian rhythms from flies to human. Nature.

[B8] Tousson E, Meissl H (2004). Suprachiasmatic nuclei grafts restore the circadian rhythm in the paraventricular nucleus of the hypothalamus. J Neurosci.

[B9] Reppert SM, Weaver DR (2002). Coordination of circadian timing in mammals. Nature.

[B10] Rivkees SA, Mayes L, Jacobs H, Gross I (2004). Rest-activity patterns of premature infants are regulated by cycled lighting. Pediatrics.

[B11] Glass L (2001). Synchronization and rhthmic process in physiology. Nature.

[B12] Lewy H, Naor Z, Ashkenazi IE (1999). From ultradian to infradian rhythms: LH release patterns in vitro. Chronobiol Int.

[B13] Lunshof S, Boer K, Wolf H, van Hoffen G, Bayram N, Mirmiran M (1998). Fetal and maternal diurnal rhythms during the third trimester of normal pregnancy: outcomes of computerized analysis of continuous twenty-four-hour fetal heart rate recordings. Am J Obstet Gynecol.

[B14] Visser GH, Goodman JD, Levine DH, Dawes GS (1982). Diurnal and other cyclic variations in human fetal heart rate near term. Am J Obstet Gynecol.

[B15] Patrick J (1982). Influence of maternal heart rate and gross fetal movements on the daily pattern of pattern of fetal heart rate near term. Am J Obstet Gynecol.

[B16] Patrick J, Campbell K, Carmichael L, Natale R, Richardson B (1982). Patterns of gross fetal body movements over 24-hour observation intervals during the last 10 weeks of pregnancy. Am J Obstet Gynecol.

[B17] Seron-Ferre M, Ducsay CA, Valenzuela GJ (1993). Circadian rhythms during pregnancy. Endocr Rev.

[B18] D'Souza SW, Tenreiro S, Minors D, Chiswick ML, Sims DG, Waterhouse J (1992). Skin temperature and heart rate rhythms in infants of extreme prematurity. Arch Dis Child.

[B19] Reppert SM, Weaver DR, Rivkees SA, Stopa EG (1988). Putative melatonin receptors in a human biological clock. Science.

[B20] Hao H, Rivkees SA (1999). The biological clock of very premature primate infants is responsive to light. Proc Natl Acad Sci USA.

[B21] Dimitriou G, Greenough A, Kavvadia V, Mantagos S (1999). Blood pressure rhythms during the perinatal period in very immature, extremely low birthweight neonates. Early Hum Dev.

[B22] Ardura J, Andres J, Aldana J, Revilla MA, Aragon MP (1997). Heart rate biorhythm changes during the first three months of life. Biol Neonate.

[B23] Updike PA, Accurso FJ, Jones RH (1985). Physiologic circadian rhythmicity in preterm infants. Nurs Res.

[B24] Bueno C, Diambra L, Menna-Barreto L (2001). Sleep-Wake and Temperature Rhythms in Preterm Babies Maintained in a Neonatal Care Unit. Sleep Research Online.

[B25] Schimmel M, Waterhouse J, Marques MD, Weinert D (2002). Circadian and Ultradian Rhythmicities in very premature neonates Maintained in Incubators. Biol Rhythm Res.

[B26] Korte J, Wulff K, Oppe C, Siegmund R (2001). Ultradian and circadian activity-rest rhythms of preterm neonates compared to full-term neonates using actigraphic monitoring. Chronobiol Int.

[B27] Weinert D, Sitka U, Minors DS, Waterhouse JM (1994). The development of circadian rhythmicity in neonates. Early Hum Dev.

[B28] Warner RM (1998). Spectral Analysis of Time-series Data.

[B29] Russell RJ (1985). Significance table for the result of fast fourier transformations. British Journal of Mathematical and Statistical Psychology.

[B30] Thomas KA (1991). The emergence of body temperature biorhythm in preterm infants. Nurs Res.

[B31] Ardura J, Gutierrez R, Andres J, Agapito T (2003). Emergence and evolution of the circadian rhythm of melatonin in children. Horm Res.

[B32] Wakayama K, Ogawa T, Goto K, Sonoda H (1993). Development of ultradian rhythm of EEG activities in premature babies. early Human Development.

[B33] Mirmiran M, Bernardo L, Jenkins SL, Ma XH, Brenna JT, Nathanielsz PW (2001). Growth, neurobehavioral and circadian rhythm development in newborn baboons. Pediatr Res.

[B34] Mirmiran M, Baldwin RB, Boeddiker M, Ariagno RL (1999). Development of circadian rhythms in premature infants. Sleep Research Online.

[B35] Thomas KA (2001). Biological rhythm development in preterm infants: does health status influence body temperature circadian rhythm?. Res Nurs Health.

[B36] Mortola JP, Seifert EL (2000). Hypoxic depression of circadian rhythms in adult rats. J Appl Physiol.

[B37] Bishop B, Silva G, Krasney J, Salloum A, Roberts A, Nakano H, Shucard D, Rifkin D, Farkas G (2000). Circadian rhythms of body temperature and activity levels during 63 h of hypoxia in the rat. Am J Physiol Regul Integr Comp Physiol.

[B38] Sharp FR, Bernaudin M (2004). HIF1 and oxygen sensing in the brain. Nat Rev Neurosci.

[B39] Chilov D, Hofer T, Bauer C, Wenger RH, Gassmann M (2001). Hypoxia affects expression of circadian genes PER1 and CLOCK in mouse brain. Faseb J.

